# The lived body (*Der Leib*) as a diagnostic and therapeutic instrument in general practice

**DOI:** 10.1007/s00508-021-01911-1

**Published:** 2021-07-23

**Authors:** Wolf Axel Langewitz

**Affiliations:** Dept. Psychosomatic Medicine, Communication in Medicine (Head: Prof. Sabina Hunziker, MD), University Hospital and University Basel, Hebelstrasse 2, 4031 Basel, Switzerland

**Keywords:** Doctor-patient relationship, Vital drive, Burnout syndrome

## Abstract

Based on vignettes from clinical cases, supervision and Balint groups this article presents a neo-phenomenological perspective on the lived experience of healthcare professionals in interactions with patients and relatives. Specifically, the familiar phenomenon of “something in the air” between two persons will be analyzed.

Constellations and situations are presented as fundamental and generic (ontological) categories that can be differentiated to understand the details and the whole (die Gestalt) of an interaction.

The term atmosphere is introduced to investigate the material carrier of something that “colors the air” between healthcare provider and patient.

The neo-phenomenological taxonomy of the lived body (der Leib) is used to describe the recipient structure of atmospheric mood.

Finally, the potential of these concepts for a more comprehensive diagnosis and for therapeutic use in general practice will be elucidated.

The goal of this article is to present a philosophical system that is universal and yet sufficiently precise to inform psychosomatic medicine in general and to build bridges between different disciplines of clinical work and theoretical reflection. The terms used in this article should be clear enough to prevent any arbitrary application. Clinical case vignettes give a first impression of the potential of this approach in differentiating patients above the level of ICD-10 (International Classification of Diseases, version 10) diagnoses and of the usefulness of paying attention to one’s own bodily experience.

The observation that something exists in between persons that goes beyond the exchange of overt behavior and outspoken messages has stimulated especially psychoanalytical thinking for quite some time. Freud is held to have discovered the importance of transference and countertransference in early writings [1]. From then on these concepts underwent various changes [2], which makes it difficult to refer to transference/countertransference phenomena as a concept with a shared understanding in the psychoanalytical community let alone of lay people. As regards the specific focus of this article, a relevant issue that needs clarification relates to bodily perceptions as the basis of transference and countertransference. In doing so, it seems wise to refer to German-speaking literature because in German the fundamental differentiation between *Leib* (the lived body) and *Körper* (the physiological and anatomical body) is possible. A recent textbook [3] deals with bodily aspects of the psychoanalytical situation, presenting many interesting aspects, but lacks one fundamental clarification: what do the authors have in mind, when they use terms like ‘*Soma, Psycho-Soma*’, *körperliches Empfinden* [4] or *leibliche Verkörperung* (a manifestation in the lived body of something originating from the corporeal body) [5]? We do not know. This article therefore tries to argue for a clearly defined differentiation of the lived and the corporeal body from a neo-phenomenological standpoint. This concept is applied to everyday phenomena in the clinical practice of doctors from so-called somatic disciplines.

## Two examples of so-called burnout

A 56-year-old female patient, who works in the IT department of a pharmaceutical company, presents with subjective fatigue and asks for a sick leave certificate, she thinks she may have depression. In the consultation, she brings up the term burnout syndrome very soon. She is unable to concentrate, starts many activities at the same time and is unable to finish them. Especially in the nights from Sunday to Monday, she has problems sleeping. All the unfinished things in her agenda would be buzzing around in her head. It turns out that she carries a burden of 12,417 unanswered mails. A pile from which now and again single furious reminders rattle down on her desk. Her speech is hectic, she has problems listening, has tried everything only to discard each one after a short time; she once took antidepressants but to no avail, has tried Yoga and Tai Chi for 2 weeks, but that was too exhausting.

A 48-year-old man, a teacher at a vocational college, with a moderate depressive episode (ICD-10 F.32.11) is admitted to a GP with psychosomatic specialization; 9 months ago he reduced his workload to 60%; however, this provided little relief, much to the contrary. He has been on 100% sickness leave for the past 3 months. On the one hand, it was OK to not have to work anymore; on the other hand, he was unable to get enthusiastic about anything (playing the piano, hiking, seeing friends).

His decline in performances was preceded by his taking on the additional task of coordinating the lesson plan about 1 year ago. His teaching hours were not reduced to compensate for the additional workload as had been originally planned. More and more often, he went to class feeling guilty because he felt that he was not properly prepared.

If while reading these short case vignettes, you add either a hectic or a lethargic prosody with the corresponding facial expressions and gestures, you realize how different the ‘atmospheric mood’ felt in these two consultations. This mood could certainly be described in descriptive adjectives; however, it could also lead to other reactions in the healthcare provider in response to the patient. It seems likely that the patients who were both labelled as ‘burnout patients’ would stimulate quite different movement impulses in the provider. The female patient will probably stimulate a calming gesture like putting the hand on her arm with the intention to slow her down whereas the male patient might provoke a stimulating gesture to get him going. In addition, the professionals’ behavior after the patients leave the consultation room will be different: when the female patient leaves the room, one feels tempted to air the room to release the charged atmosphere. When the male patient leaves, the energy level will be lower and the healthcare professional might stretch his or her muscles and bend the arms to wake up the numb muscles.

We could now postulate that something in the way these two different patients present themselves spreads towards the healthcare provider, or “coloring” the atmosphere in a specific way, thus having an impact on prosody, movement impulses and possibly one’s own sense of (dis)comfort. These impulses must not necessarily be acted out; it would be enough to sense them as an intention or an inner need. What we are referring to is a ‘bodily response’ to something specific in these patients. Starting from the two hopefully plausible examples above, the following aspects will be discussed in more detail in the following:

- Which fundamental ontological categories offer a framework in the search for explanations—situation and constellation as basic manifestations of the world.
- What is ‘coloring the air’ between healthcare provider and patient—the power of atmospheres.
- How can the atmospheres affect a health care professional—a theory of the lived body (Leib).
- How could all this apply to medical practice?

## Situation and constellation

An interesting question is in which “world order” one should start searching for answers. As representatives of a discipline that views itself as being primarily based on the natural sciences, physicians tend to consult the empirical literature to identify phenomena that describe in detail what happens between and with two people, who in some way influence each other or, metaphorically speaking, ‘rub off’ on each other. This search leads to interesting empirical findings within a scientific model, a few of which are presented. They apply to the empirical basis for mutual recognition and understanding and ensuing recommendations for empathic behavior.

In the case descriptions, patients are characterized by different modes of movement, prosody, and facial expression, relating to overt behavior. Researchers have been especially interested in describing and manipulating body movements or facial expressions in performing artists who might be seen as extraordinarily proficient in reaching out to other persons (the audience) via their professional mastering of bodily expressions. One exemplary paper shows that observers accurately identified the intended emotional expression in neutral sentences sung or spoken by an artist from the specific pattern of head movements and postures [6]. The authors use the term ‘encoding’ to describe the generation of an emotional impression that corresponds to the artist’s intentions. They conclude: “These results provide the first evidence that head movements encode a vocalist’s emotional intent, and that observers decode emotional information from these movements.” In another paper the authors investigated whether bodily signals are more important than changes in facial expression to identify intense negative or positive emotions [7]. They concluded that high intensity emotions are better identified from body movements than from changes in facial expression. Assuming that correct identification of the other person’s emotional state is a prerequisite for a successful interaction between two persons [8, 9] other researchers were interested in defining the neurobiological basis for such an interpersonal agreement. In these studies, ‘mutuality’ is searched for in brain activity. A very elegant and recent study used functional magnetic resonance imaging (MRI) to demonstrate how ‘two brains’ interact in an empathic way. They reported that in patient-clinician dyads with high pre-test empathy ratings the brains of partners showed highly coupled activity [10].

Reading these articles one big question arises. Put in somewhat polemic terms: what do I know about my brain? Of course, given my professional background, I assume that it is active in some way while I write and while you read, but I certainly could not say what exactly is taking place up there. The articles cited above are certainly very interesting, but I would posit that they report on mere correlates of what study participants describe as their own perceptions. Whether or not a person is affected by someone else and whether or not this has an influence upon this person’s momentary feelings, is a fundamentally subjective fact that can only be alluded to by this very person in this very moment or from vivid memories. The biological processes that are taking place at the same time, however, are not accessible as such.

In my view, papers recommending a certain set of behaviors to make healthcare professionals more empathic, also suffer from the same shortcoming by suggesting that if they pay attention to a list of communication means, they will be in command of their empathy. A paper with the telling title E.M.P.A.T.H.Y. mentions E: eye contact; M: muscles of facial expression; P: posture; A: affect; T: tone of voice; H: hearing the whole patient: Y: your response [11]. This and similar papers parse a comprehensive phenomenon like the vivid (in the sense of creating a certain resonance) perception of the other into a set of single components. I would posit, however, that only the composite Gestalt of these elements is capable of conveying a reliable impression of the other and indeed, there is no doubt that humans have the capability to judge another person quickly without paying attention to circumscribed elements. This has been shown, for example, in papers by Naumann et al. [12], in which certain personality characteristics were identified with high precision.

Neo-phenomenology, a philosophical system founded by Hermann Schmitz [13], stresses the importance of the fundamental distinction between a world view that consists of a complex array of single elements on the one hand, and a world view consisting of a comprehensive impression, like the perception of a Gestalt, on the other hand. In neo-phenomenological terms, this distinction refers to the difference between *constellation* and *situation* (for an application see [14]).

Hermann Schmitz chose the example of a person using his or her mother tongue, as opposed to someone who wishes to learn a new language, to illustrate the difference between a situation and a constellation [15]. We usually find it easy to express ourselves in our mother tongue without knowingly applying grammatical rules or consciously choosing certain words from the rich reservoir of our vocabulary. Rules and words are contained in the situation of our mother tongue in chaotic multiplicity; however, we do not lose orientation. In a foreign language that we have not yet mastered, we must remember certain grammatical structures, like how to phrase a question in French, and we deliberately pick single words that we think would fit in a certain context. This is a consciously controlled process, in which single elements are arranged in a certain albeit highly complex order, thus forming a constellation.

If we wish to describe the experience of being subjectively involved by another person (*Betroffenheit*), we could refer to the category of a constellation and identify single elements of the interaction like in the E.M.P.A.T.H.Y paradigm quoted above. Otherwise, we might try to grasp meaning from the whole of a situation that is impossible to describe in single terms but dissolved in chaotic multiplicity. Especially, in general practice, this vague yet often prominent sense of ‘something is wrong’ (‘with me’ from the patient’s perspective or ‘with this patient’ from the doctor’s perspective) is a familiar phenomenon, most often found in patients with so-called multiple unexplained symptoms or somatization or somatic symptom disorder [16].

## The power of atmospheres

In this section an attempt is undertaken to answer the question: what is it that ‘colors the air’ between a care provider and a patient giving rise to the phenomenon of subjective involvement? These considerations start from the common experience of ‘something in the air’ (not necessarily always love like in the famous song by John Paul Young) that adds a specific flavor to the climate in a room. It could be a closed room which one enters and has the immediate impulse to draw back (‘trouble is brewing!’) or a wide landscape that opens the heart. We have all sat in an audience sensing the joyful expectation before a star soloist enters the stage or could at least imagine the tense silence before a senior manager explains the consequences of a prolonged lockdown to a group of employees.

These familiar phenomena are reflected in the theory of feelings in neo-phenomenology. Feelings are not necessarily a private affair, encapsulated in the inner self of humans, but very much capable of radiating into the environment, or creating an atmosphere where other human beings (and social animals like dogs, cats, or horses) are affected [17, p. 343]. This then generates a responsive behavior (e.g. in animals) or might even cause corresponding feelings in a sensitive other. Feelings are understood as atmospheres that have no clear margin, no rim. Therefore, one is unable to say where exactly a sense of ‘trouble is brewing’ starts, or in other words, where a threshold is located that divides a neutral from a tense atmosphere in a given room.

This immersion into a certain atmosphere is not only sensed by a healthcare professional when a patient enters the consultation room or when a doctor enters a room where patient and family are waiting to hear bad news. It is also felt by patients, who sometimes make comments like: “as soon as I entered her consultation room, I immediately felt at ease”. Architects try to promote a positive, caring or reassuring atmosphere when they design the interior of practices or hospital buildings [18].

Feelings as atmospheres are not always or necessarily felt vividly by individuals; sometimes individuals just recognize a certain atmosphere without being affected themselves. It is only through the experience of being moved by an atmosphere that an individual is able to sense a feeling as if it was his or her own. Being moved by an atmosphere is a sensation of the lived body (der *Leib*) as the famous quotation from Gretchen in Faust I exemplifies: “A shudder ran through my whole body (“*Mir läuft ein Schauer über’n ganzen Leib*”)” [19verses 2755 and 2757]. A specific characteristic of working with atmospheres is that it is impossible to identify the source of a certain mood with absolute certainty.

### A sense of anxiety and guilt

In a supervision group, a participant reports on the following situation: He had (again) sensed a resistance in the left breast of a 42-year-old patient. Even though she had a long-standing history of fibroadenoma of the breast, he performed an ultrasound ‘for safety reasons’, he said. He discovered a suspicious structure below the node and upon scanning the whole breast, several other structures of unclear identity. He then initiated all necessary steps at a certified breast cancer center, where a histological sample was taken. An appointment was made in 5 days to inform her about the results. Over the weekend, the husband asked for an earlier appointment per email; however, without the results of the histological findings, this made little sense and he declined. When he saw the couple waiting to be taken into the consultation room, he suddenly felt insecure, almost attacked by a sense of shame because he thought he had forgotten another follow-up appointment (which later he discovered in the patient’s charts). During the encounter, he felt anger and intense feelings of reproach. The husband’s frenetic activity stood in contrast with the surprisingly calm goal-oriented reasoning from the patient.

In the reflection of this episode, the doctor interpreted the sense of reproach as being directed towards himself, he could only think of himself as the source of the husband’s unease. When asked to describe his personal bodily experience, he reported about a dragging sense of narrowing, most prominent in his stomach and his chest, problems breathing deeply, and a sense of warmth in the forehead. Other participants in this group associated these descriptions—in the light of the case report—as being indications of shame and guilt, of a sense of being cornered and of anxiety. Once these associations were taken as phenomena inherent to the *shared situation* between doctor, patient and husband without assuming just one underlying direction, from husband towards the doctor, it became evident to the group that the very situation between patient and husband could have given rise to exactly the same feelings: self-reproach (“how could I ever ignore these changes in my breast?”), accusations from the husband towards his wife (“how could this happen? You always said you know yourself best and examine yourself carefully”, etc.), a sense of being cornered by an evil fate, narrowing in on the fight against cancer, etc. became as likely as the primary associations of the colleague who had presented the case.

When it is possible to describe the specific mood of an atmosphere in such a vivid and precise way, sensing one’s momentary bodily state (‘body’ as the felt or living body *Der Leib*) turns into a diagnostic instrument. If furthermore, the professional is able to talk about bodily phenomena with a patient or relative in such a ‘non-directional way’, as something that is simply there and can be understood in several ways, this might increase the degrees of freedom in the interpretation of a given situation and thus gain in therapeutic potential.

## A theory of the lived body (*Der Leib*) in neo-phenomenology

In the previous sections, the term ‘lived body’ has often been used without a precise explanation. This deficit will now be addressed in more detail to answer the question where atmospheres are felt.

Contrary to Roman languages or to the English language, German offers two different terms, namely the term *Leib* (felt or lived body) and *Körper* (physiological or anatomical body or corporeal body). These terms are not interchangeable, as the example of the ‘*Leibarzt’* denotes which could not be transformed into a ‘*Körperarzt’*. Likewise, the Latin “*hoc enim est corpus meum*” is translated as “*Das ist mein Leib* (this is my lived body)”, whereas the English language uses the term ‘body’ without any further differentiation.

Sensations in the lived body (*der Leib*) are characterized by a less precise localization compared to sensations of the corporeal body (*der Körper*). This is illustrated by the difference between an inner sense of anxiety or being scared, as opposed to pain in the knee after a long walk, or pleasant warmth in the feet with a hot water bottle in bed. The latter two perceptions are easy to locate with high precision, we might be able to say ‘it is somewhere inside my knee joint and it hurts more if I stretch the leg’, etc. The notion of being scared and anxious, the apprehension of impending doom is of a different quality. In most people, the sensation will be most prominent in the abdominal region; however, it is much more difficult to localize compared to the pain or warmth in the examples above [20].

This difference in orientation in space is denoted in the following definition:

- A phenomenon relates to the physiological or anatomical body, if it can be found in a distinct place: a patient can easily point to the location of a swelling; likewise, the professional can see and touch it at the same location.
- A phenomenon relates to the lived body if sensed in a region of the physiological or anatomical body without using any of the five senses to locate it [21].

The capacity to interact with other persons as carriers of their lived body underlies the ability of most people to move through a busy city center without bumping into someone else. It reflects their implicit ‘knowledge’ of the dimensions and the range of movement of their felt body. This knowledge does not result from a deliberate step by step adjustment of one’s expansion and speed of moving forward. Instead, it results from a spontaneous tendency to form a shared body that encompasses the other persons’ and the own lived body via something that could be called mutual embodiment [21pp. 29].

This ability to spontaneously form a shared body with other persons or with objects in the environment is prone to irritations: wearing a new wristwatch with a slightly thicker body leads to unwanted contact with the doorframe, until the new watch becomes ‘embodied’, i.e. an integral part of the lived body. The same holds for a musician for whom the instrument is an extended part of his lived body or a sportsman who has embodied, e.g. his tennis racket. The assumption of ‘mutual embodiment’ as the source of the smooth passing by of people in the crowd of Christmas shoppers is also strengthened by those moments in which this mutual orientation fails. This is typically the case when a person is ‘embodied’ with his or her mobile phone, instead of with other members of the crowd.

## Theory of the lived body and perception phenomena in medical practice

Hermann Schmitz has developed an elaborate alphabet of the lived body allowing for a precise description of bodily (*leiblich*) phenomena and their identification in daily life [21, pp. 15, 22, p. 74, 23]. The two patients described at the beginning of the article who both were designated as suffering from burnout, might serve as an opportunity to illustrate the usefulness of the concepts of neo-phenomenology for medical practice. Apparently, these patients differ in a specific aspect of their bodily (*leiblich*) appearance. One striking aspect relates to a difference in their perceived vitality: whereas the female patient induces a vibrant or rather hectic atmosphere, the male patient seems dull. This vitality is not necessarily linked to a healthy young corporeal body but might also be present in a ‘vital’ elderly person, who in spite of advanced age appears energetic and interested in life. According to Schmitz, this vitality is a manifestation of the lived body, not the result of objective physiological or anatomical dimensions of the corporeal body (*Körper*).

In order to understand the origin of vitality, the so-called vital drive, it is necessary to identify the driving force within the lived body. According to neo-phenomenology, this can be traced back to the antagonism between a narrowing and a widening tendency in the lived body. Referring to everyday experiences, a narrowing tendency, most often in the abdomen, is apparent when, e.g. one steps into the void because the end of the stairs is still one step lower. A widening tendency is typically felt in the breast after a deep breath of fresh air. Vital drive results from the antagonism of narrowing and widening tendencies: using the metaphor of two teams pulling a rope with the rope standing for the vital drive, the rope is even more loaded with energy the more the two teams pull in opposite directions. If one side lets go, the rope falls down, energy collapses.

To some extent, vital drive is not amenable to conscious control at the disposition of an individual. One might think of the paradigmatic nervousness of an Arabian thoroughbred and the unflappability of a heavy horse drawing a brewery carriage. A heavy horse will never be able to run as fast as a thoroughbred, and the elegant Arabian will not be able to pull heavy loads. The latter example denotes the idea of a bodily disposition as a constituent quality of a living being: individuals differ in their ability to build up and maintain vital drive, most clearly in animals but certainly in humans, as well. If someone can meet high demands only by straining all his or her power, vital drive will eventually be exhausted.

Vital drive, however, does also undergo temporary changes: long-standing reduction of vital drive cannot only result from chronic mental or physical illness but also from a mismatch between the necessity to be alert (‘always on call’) as a parent or as a professional with high responsibility and the ability of the lived body to hold up the antagonism between narrowing and widening tendencies. Going back to the clinical examples, one might speculate that first both patients differ fundamentally in their bodily disposition with the manager having a higher vital drive than the teacher and that second, in both patients a mismatch exists between the capacity of their vital drive and the professional or private demands. Furthermore, the hectic female patient has a problem with the assignment of her vital drive to certain tasks; she is no longer able to focus her high gear vitality; her energy is split among too many demands and therefore loses momentum. It is very likely that after a while ‘many hounds will soon catch the hare’—she is too busy in too many fields and this will exhaust even this energetic woman.

For this patient, the problem is not a low overall level of vital drive but her inability to turn her vital drive towards single tasks. Accordingly, a reduction in the multiplicity of demands during vacation led to a temporary improvement of her situation: she managed to keep afloat, to judge workload correctly, to delegate tasks to others, to abstain from checking her mail account on Sunday, etc.—she was able to bundle her forces. During therapy, she found readjusting her workload extremely difficult. This was partly due to the hurtful recognition of her position within her company’s hierarchy. She was not in a managerial position where distribution of workload is a major issue, where she would have others working for her, but in a position where her duties were a mixture of a high load of executive functions and a small chance to lead. Furthermore, her family background was characterized by the importance of ‘not being lazy’ in the sense of: a good girl always knows what to do next.

She found it difficult to carefully pay attention to her bodily state, a reduction in hectic activities was equated with failure and boredom. This general belief was well in line with the Zeitgeist that praises continuous acceleration [24] as the primary source of a society’s welfare.

In the male patient, the restitution of his reduced vital drive was a more difficult and lengthy task characterized by many setbacks. Based upon the basic notion that vital drive results from the antagonism of narrowing and widening tendencies in the felt body, therapy was primarily focused on the identification of moments in which both tendencies were present. One such moment was the close observation of phenomena that were present during the shower in the morning. Will he turn off the water when there is no more shower gel on his body or shampoo in his hair? Or does taking a shower come to an end when the sense of the warm water pouring down on him causes no more delight but becomes dull? What happens when he turns the cold water tap on?

When he had achieved a higher sensitivity for the perception of his bodily state, he became able to arrange real life experiences and desires within the framework of narrowing and widening tendencies with the idea of finding a better balance between them. After 5 months, a decisive moment was a trip to the Lofoten Islands that he had dreamed of for a long time and had never managed to take. He vividly recalled a walk uphill, against chilly winds with cold rain in the face, sensing the warmth on his skin below the anorak and in his legs, making their way up. His descriptions of sipping hot tea from his thermos flask at the windy summit and the sense of immersion into the hot water in the bathtub back at his hotel were especially impressive. The stepwise resumption of his professional activities was accompanied by a careful balancing of narrowing and widening experiences in his daily life.

Along the same vein, paying attention to one’s bodily state is a prerequisite for professionals who wish to make use of the chances that mutual embodiment with a patient can offer. Patients often use metaphors or terms that originate in bodily (*Leiblich*) phenomena, they sometimes re-enact what occurred to them when they talk about a specific event that they wish to bring to the attention of the professional. Therefore, the raw material to work with is available, providing professionals are willing to take what a patient is relating literally. This is even more rewarding if professionals not only encourage patients to talk about their feelings but also pay attention to the atmospheric mood. Not so rarely, someone talks about being sad but no sadness can be felt in the lived body of the observer. On the other hand, we all remember situations in which a funny story is told and we do not feel amused but sense a certain heaviness, we choke on our laughter. In that case, the atmospheric mood contradicts the verbal content of what has been said. In a previous article, we pointed to the importance of the difference between feelings expressed verbally and feelings sensed bodily especially in Balint groups [25].

It should, however, also be mentioned that working with bodily awareness also carries a certain risk. The following example from a Balint group session shows that an intensely loaded atmosphere has the potential to cast a spell on the group and paralyze the group facilitator.

### A paralysed Balint group facilitator

A psychiatrist talks about a quirky retired teacher, who lives alone, has no contact with anybody except for the psychiatrist whom he sees at monthly intervals. This patient spreads a funereal atmosphere; she feels her heart getting heavy whenever she sees him. When he leaves, she would always feel the need to open the windows to let in fresh air. Heaviness weighs on the group and on the facilitator; the speaker does not make the situation easier when she suddenly recalls that the teacher had told her about bags full of weed killer in the cellar, enough to kill the population of the whole village. He had once rather casually mentioned this fact and asked whether she had an idea how he could best get rid of this very special treasure. The group remains silent, the facilitator is lost in thought, everybody is brooding in unbearable heaviness.

What is missing here is a participant (or perhaps the group facilitator?) who manages to resist the undertow of the shared situation and to raise his head above the muddy broth to sort out the facts with a clear view. If someone in this group had been able to distance himself from the heavy atmosphere and focus on single facts instead, this participant had switched from a situation into a constellation. In this world of single elements, it would be interesting and certainly relevant to know whether the situation is likely to develop into a substantial danger for the public, for the patient himself, or for the presenting colleague (accusation of negligence?).

If atmospheric mood is given an opportunity to develop, then not only heaviness but also its opposite can be impressive. If we think of a patient who through her bodily (*leiblich*) constitution is transmitting a shining lightness, a professional might be tempted to act more carelessly than normal, not taking bodily complaints as seriously as in other patients—everything feels so easy going and nothing can happen, gravity is out of function.

## Resume

I hope these reflections can contribute to a greater awareness of the potential of bodily perceptions in healthcare providers and patients in everyday practice in medicine. I would argue that bodily phenomena of the kind described in this article, are so universal and familiar to human beings that healthcare providers could add them to the repertoire of their diagnostic tools. At the very least, they might recognize the richness of their personal participation in an encounter with a patient, they might realize that they are healthcare professionals with heart and soul. A change in the atmospheric mood tells us something about the inner world of the patient and/or about the shared situation of healthcare provider and patient. If it is possible to address these felt changes, talking about them may even have therapeutic potential, even if it only encourages the patient to take what is happening to him seriously.
